# Correction of “Wrist” Deformity in Radial Dysplasia: A Systematic Review and Meta-Analysis

**DOI:** 10.2106/JBJS.17.00164

**Published:** 2017-12-20

**Authors:** George R.F. Murphy, Malcolm P.O. Logan, Gill Smith, Branavan Sivakumar, Paul Smith

**Affiliations:** 1 Department of Plastic and Reconstructive Surgery, Great Ormond St. Hospital for Children, London, United Kingdom; 2 Randall Division of Cell and Molecular Biophysics, Guy’s Campus, King’s College London, London, United Kingdom; 3 The Portland Hospital for Women and Children, London, United Kingdom

## Abstract

**Background:**

Radial dysplasia affects 1 in 6,000 to 8,000 births, classically presenting with a shortened, bowed ulna and radially deviated hand. The optimal treatment remains unclear, with several opposing approaches advocated. This review aims to clarify the long-term outcomes of nonsurgical and surgical treatment of the “wrist” deformity.

**Methods:**

The Embase, MEDLINE, PubMed, Cochrane Central, ClinicalTrials.gov, and World Health Organization International Clinical Trials Registry Platform (ICTRP) databases were searched for published and unpublished studies reporting long-term outcomes of surgical or nonsurgical treatment of children with radial dysplasia. Results were not restricted by date or language. Primary outcomes were hand-forearm angle, ulnar length, and “wrist” active range of motion (ROM). Studies were assessed using the Grades of Recommendation, Assessment, Development and Evaluation (GRADE) criteria. Data for the change in hand-forearm angle were pooled using random-effects meta-analysis, and mean differences and 95% confidence intervals were obtained. Primary outcome data at last follow-up were pooled, and means and standard deviations were obtained. The PROSPERO registration of this study was CRD42016036665.

**Results:**

Of 104 studies identified, 12 were included in this review. Five were retrospective cohort studies and 7 were case series. No randomized studies were found. Study quality was low or very low according to the GRADE criteria. The hand-forearm angle of nonsurgically treated patients worsened during childhood, from 66° to 84°, whereas “wrist” active ROM, at 61°, was better than that for most surgically treated patients. Ulnar length with nonsurgical treatment was predicted to be 64% of normal, but was not directly reported. Isolated soft-tissue release provided a modest reduction in hand-forearm angle compared with nonsurgical treatment. Soft-tissue distraction with centralization or radialization achieved the best hand-forearm angle correction (16° radial deviation). Radialization maintained better “wrist” active ROM (46°) and ulnar length than centralization. Microvascular second metatarsophalangeal joint transfer yielded better reported “wrist” active ROM (83°) and good ulnar length compared with other surgical techniques, but a slightly worse hand-forearm angle (28°).

**Conclusions:**

There was low-quality evidence that soft-tissue distraction plus centralization or radialization achieved the best correction of the hand-forearm angle for children with radial dysplasia.

**Level of Evidence:**

Therapeutic Level IV. See Instructions for Authors for a complete description of levels of evidence.

Radial dysplasia, also known as “radial longitudinal deficiency,” includes “radial clubhand” and is a disfiguring, and potentially disabling, congenital limb anomaly (Figs. 1-A and 1-B). The entire upper limb may be involved, although the defect is most evident in the forearm and hand^1^. Affected children suffer a variable degree of hypoplasia or absence of the preaxial skeleton and soft tissues, in particular the thumb, radius, and dorsoradial soft tissues. The hand is usually radially deviated and subluxated off the distal aspect of the ulna, the ulna may be shortened and have a bow-shaped deformity, and there is no true wrist (radiocarpal) joint in Bayne^2^ type-III and IV radial dysplasia. The incidence in methodologically sound whole-population studies is 1 in 6,000 to 8,000 live births^3-5^; this is an order of magnitude higher than the classical estimates^6^ of 1 in 30,000 to 100,000, derived from specialist clinic referrals that may have missed patients who were referred elsewhere, treated locally, or not treated at all. The same whole-population studies report perinatal mortality approaching 30%, possibly from associated anomalies, reducing the number of affected individuals in the general population to about 1 in 10,000.

**Fig. 1-A:**
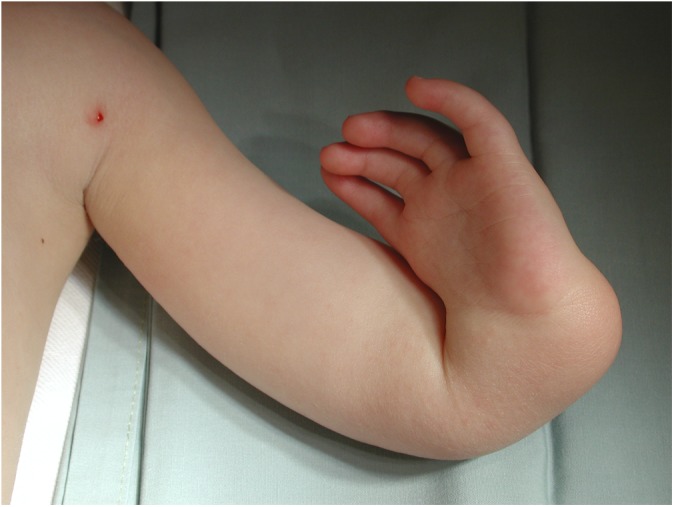

**Fig. 1-B:**
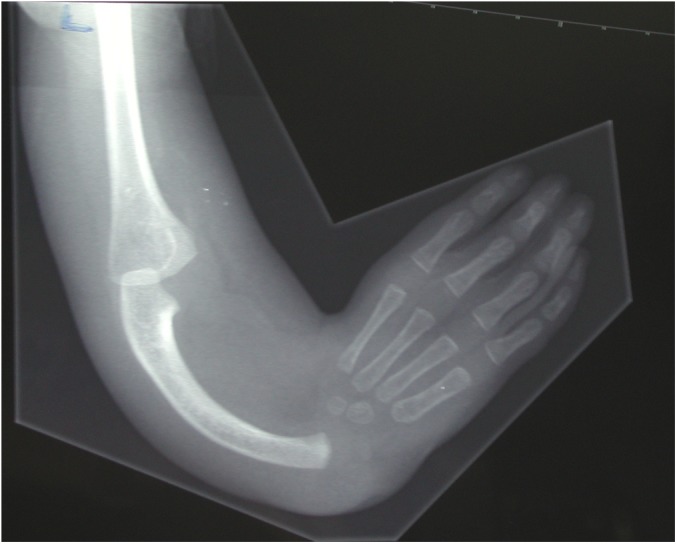
**Figs. 1-A and 1-B** The left upper limb of a child with unilateral radial dysplasia, before surgery.

Known causes include spontaneous mutations, teratogenic drugs, and syndromes such as Holt-Oram, VACTERL (vertebral, anal, cardiac, trachea-esophageal, renal, and limb defects), or Fanconi anemia, although currently <30% of patients receive a confirmed genetic diagnosis^7^. The most common genetic causes are summarized in Figure 2. Associated syndromic malformations include cardiac, renal, and vertebral malformations and blood dyscrasias; as these may be asymptomatic at referral, most centers screen patients with radial dysplasia for these routinely.

**Fig. 2:**
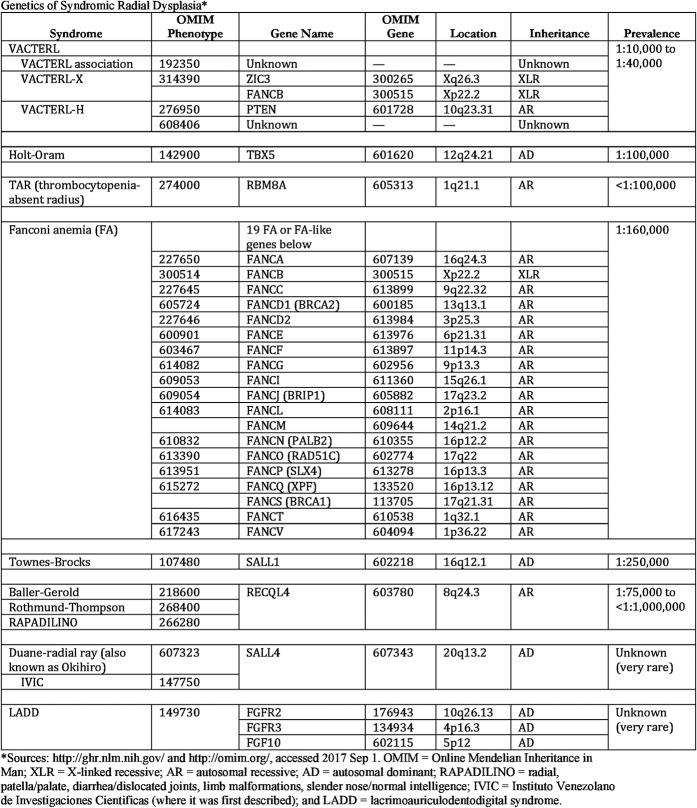
Genetics of syndromic radial dysplasia.

The best treatment for radial dysplasia is unclear, with several opposing approaches currently advocated as optimal. Options include nonsurgical treatment^8-11^, isolated soft-tissue release^12,13^, repositioning the carpus on the distal aspect of the ulna by centralization^6,14^ or radialization^15^, and radial substitution with imported tissue, such as microvascular transfer of the second toe^16^ or proximal fibular epiphysis. The most common approach globally involves soft-tissue distraction^17^, followed by some form of surgical centralization or radialization with adjunctive tendon transfers to reposition and rebalance the hand. However, whether treated operatively or not, these patients suffer poor forearm growth, which may be worsened by surgery, and many have some degree of recurrent radial “wrist” deviation^1,18-20^.

This systematic review aims to clarify the long-term morphological outcomes (hand-forearm angle, ulnar length, and “wrist” active range of motion [ROM]) of current treatment techniques for children with radial dysplasia, and to compare them with those of nonsurgical treatment. These outcomes were chosen because they have been widely reported, not because a well-aligned hand is necessarily “better.” It remains unclear which outcomes actually matter to patients, although a core outcome set is being developed with patient participation to address this^21^. However, our experience is that most parents seek correction of the deformity as their primary aim, even though many of these children are functionally competent to a variable degree.

## Materials and Methods

### Search Strategy

We searched MEDLINE and Embase via OvidSP (all fields), PubMed (all fields), the Cochrane Database of Systematic Reviews, and the Cochrane Central Register of Controlled Trials (searched on December 2, 2016). The ClinicalTrials.gov trial registry and the World Health Organization (WHO) International Clinical Trials Portal (http://apps.who.int/trialsearch/Default.aspx) were also searched (on December 2, 2016) to identify unpublished trials. The search strategy in Table I was developed to retrieve all studies and reviews of outcomes following surgical or nonsurgical management of radial dysplasia. Searches were not limited by date, language, or publication status. Search results were independently screened for relevance by 2 authors (G.R.F.M. and B.S.). Full-text articles were retrieved via the Bodleian Library (Oxford, U.K.) and British Library (London, U.K.). Disagreements on study eligibility were resolved by consensus, with reference to a third author if required. Study selection is outlined in Figure 3. The study protocol was prospectively registered with the PROSPERO database (http://www.crd.york.ac.uk/PROSPERO/display_record.asp?ID=CRD42016036665).

**TABLE I:**
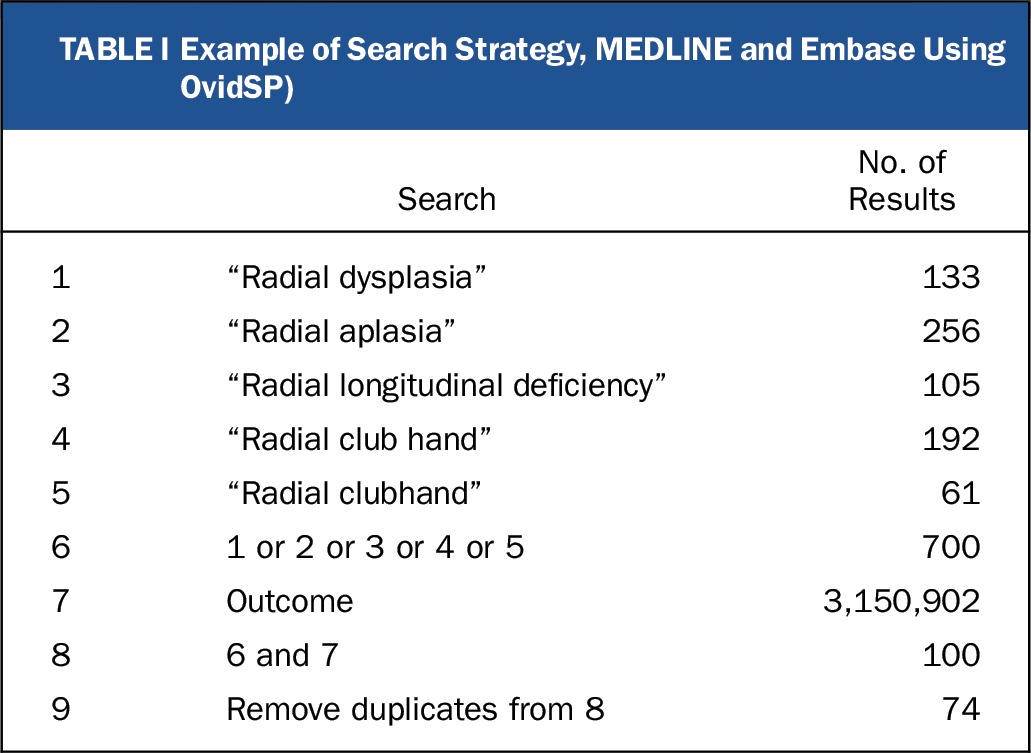
Example of Search Strategy, MEDLINE and Embase Using OvidSP)

**Fig. 3:**
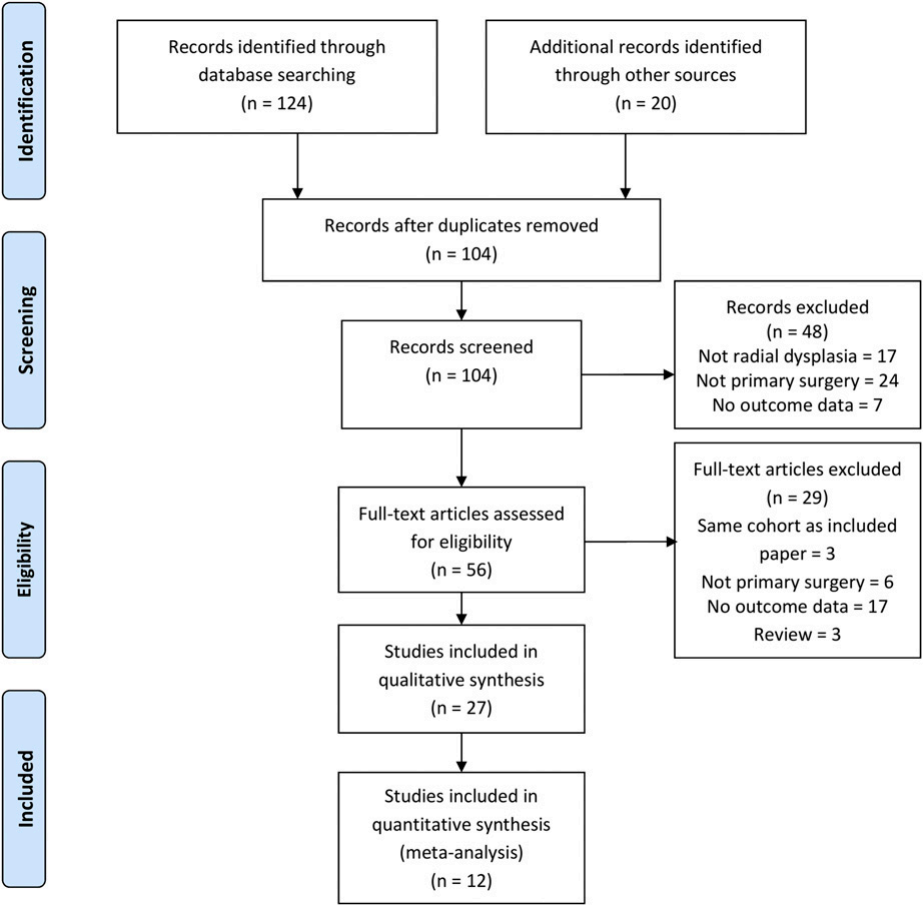
PRISMA (Preferred Reporting Items for Systematic Reviews and Meta-Analyses) flow diagram for the study.

All studies reporting patient outcomes after any form of surgical treatment or nonsurgical management for Bayne^2^ type-II, III, and IV radial dysplasia were included. Studies reporting outcomes in both adult and pediatric populations were included. Interventional and observational studies were included. Studies without quantified follow-up data after treatment were excluded. Studies with follow-up of >9 years were included in the meta-analysis; data from clinically homogenous series were pooled by operative technique. For studies included in the meta-analysis, the outcome measures were the hand-forearm angle, ulnar length, “wrist” total active ROM (all at the time of last follow-up), and change in hand-forearm angle from preoperatively to the time of last follow-up. Included papers were screened to ensure that all relevant primary studies were included.

### Data Collection and Analysis

Studies were assessed for risk of bias using the Grades of Recommendation, Assessment, Development and Evaluation (GRADE) criteria for observational studies. Two authors (G.R.F.M. and B.S.) independently extracted data on the number, disease severity, and treatment of study participants, and the mean and standard deviation for the preoperative and postoperative hand-forearm angle, ulnar length, and “wrist” total active ROM. For studies with missing data, the corresponding author was contacted to request them. Where the range was reported instead of the standard deviation, the standard deviation was estimated using the range rule: standard deviation = (maximum – minimum)/4.

Duration of follow-up is important in pediatric hand surgery, as the limb changes throughout growth, with the final result only apparent at skeletal maturity. We therefore restricted our meta-analysis to the 12 papers with a mean follow-up of >9 years, which are likely to have included a minimum of 2 major growth spurts. Studies were grouped by operative technique for the meta-analysis. For the change in hand-forearm angle (preoperative to last postoperative follow-up), mean differences and 95% confidence intervals were calculated. Ulnar length and “wrist” total ROM were infrequently reported preoperatively, so the mean and standard deviation at the last follow-up were calculated. Two authors (G.R.F.M. and B.S.) assessed the participants, interventions, and outcomes for clinical heterogeneity. Because of the variety of study methodologies and treatment regimens used, study data were pooled using a random-effects model. The low methodological quality of the original studies limited meaningful statistical comparison among the various surgical techniques that they utilized. We have, therefore, presented the results in narrative form rather than calculating significance levels. Statistical advice was provided by the King’s College London Research Support Team. Statistical analysis was performed using Excel 2016 (Microsoft) and RevMan 5.3 (Nordic Cochrane Centre).

## Results

We identified 104 studies and extracted data from 27 of these; 12 studies reported in 14 papers were included in the data synthesis (meta-analysis). Figure 3 summarizes the review process. Of the studies in the data synthesis, 5 were retrospective cohort studies reporting the outcome of >1 treatment approach, and 7 were case series reporting the outcome of a single treatment approach. No randomized studies were found. With the exception of the study by Kotwal et al.^8^ (n = 446), all were small (n ≤ 35). The methodological quality was low or very low, according to the GRADE criteria, for all studies (see Appendix).

Table II summarizes the data synthesis results (see Appendix for the full data set). Many centers use both centralization and radialization, and choose between them according to the intraoperative soft-tissue quality. We therefore calculated the combined results of centralization or radialization after soft-tissue distraction in addition to the results for the individual techniques.

**TABLE II:** Long-Term Results of Treatment, by Modality (>9 Years of Follow-up)*

				Final Results‡		
Technique	No. of Limbs (Patients)	Mean Follow-up *(yr)*	Change in HFA† *(deg)*	HFA *(deg)*	UL *(cm)*	“Wrist” AROM *(deg)*
Nonsurgical^8-10,22^	147 (109)	14.1	19.0 (13.8, 24.2)	83.8 ± 24.0	16.1 predicted	61.0 ± 26.3
Centralization^1,9-11,19,26^	103 (75)	15.3	−34.4 (−46.1, −22.7)	27.6 ± 23.4	12.2 ± 2.9	34.4 ± 25.0
SD and stabilization^8,23-25^	356 (283)	13.5	−58.0 (−71.7, −44.3)	12.6 ± 16.8	12.9 ± 3.0	40.8 ± 24.4
SD and centralization^23-25^	21 (19)	11.1	−71.4 (−93.2, −49.5)	16.7 ± 20.1	11.5 ± 2.2	25.4 ± 18.2
SD and radialization^23,24^	26 (25)	11.0	−49.1 (−63.2, −35.4)	16.5 ± 22.5	13.6 ± 3.1	46.3 ± 29.3
Soft-tissue release and bilobed flap^12,13^	18 (16)	9.2	−24.0 (−34.6, −13.4)	64 ± 13.3	16.1 ± 1.4	73
SD and microvascular 2nd MTPj transfer^27^	19 (18)	11.0	−33.7 (−46.2, −21.2)	27.9 ± 14.4	15.4 ± 2.5	83.2 ± 21.9

* HFA = hand-forearm angle, UL = ulnar length, AROM = active range of motion, SD = soft-tissue distraction, and 2nd MTPj = second metatarsophalangeal joint.

† The values are given as the mean difference, with the 95% confidence interval in parentheses.

‡ The values are given as the mean and the standard deviation.

The hand-forearm angle of nonsurgically treated^8-10^ patients worsened during childhood, from 66° to 84°, but “wrist” active ROM, at 61°, was better than for most surgically treated patients. The ulnar length of nonsurgically treated patients was not reported in the studies included in the meta-analysis. In studies of nonsurgically treated children with shorter follow-up, Heikel^11^ observed that ulnar length was one-half to three-quarters of normal, and Sestero et al.^22^ found that ulnar length was 64% of normal, predicting growth of 16 cm by an age of 15 years. The technique of isolated soft-tissue release with a bilobed skin flap, used by Vuillermin and colleagues^12,13^, resulted in only a slight improvement in the hand-forearm angle, to 64°, but achieved 73° of “wrist” ROM and ulnar length of 62% of normal (16.1 cm at an age of 17 years).

When compared with nonsurgical treatment, soft-tissue distraction with either centralization^23-25^ or radialization^23,24^ achieved the largest correction of the hand-forearm angle, to a final radial deviation of 17°. It is noteworthy that patients treated with centralization had a greater hand-forearm angle deformity to start with, so although the final angle was the same, patients treated with centralization had a greater improvement in the angle, 71°, compared with patients treated with radialization, in whom the hand-forearm improved by 49°. Radialization maintained better “wrist” ROM (46°) and ulnar length (13.6 cm) than centralization (25° and 11.5 cm). Both yielded greater hand-forearm angle correction than in historical series of centralization without prior soft-tissue distraction^1,9-11,19,26^, which achieved a mean improvement of 34° to a final radial deviation of 28°, and similar ulnar length and “wrist” ROM.

Vilkki^27^ reported better “wrist” ROM (83°), with good ulnar length (15.4 cm), after microvascular second metatarsophalangeal (MTP) joint transfer than after other surgical techniques, at the cost of a smaller improvement in hand-forearm angle (28°); these results are not surprising, as this is the only technique in the data synthesis that imports a true joint to the “wrist.” It should be noted that the hand-forearm angle in that study was recorded after “gentle stretching” of the hand, to measure the “radial tightness.” The hand-forearm angle in that series is therefore not directly comparable with those in other series, which measured the unstretched hand-forearm angle, as described by Manske et al.^28^.

Finally, it is worth noting that most series show a tendency for deviation to recur during growth, although some series indicate the achievement of durable correction despite this.

## Discussion

We believe this study to be the first systematic review of the outcomes of treatment for radial dysplasia. Our findings suggest that, over the medium to long term, soft-tissue distraction plus a stabilization procedure (centralization or radialization) provides substantially better correction of the hand-forearm angle compared with nonsurgical treatment, at the cost of some loss of “wrist” ROM and possibly also of ulnar length. Although both centralization and radialization achieved the same final hand-forearm angle, that represented a much greater change in hand-forearm angle, from a more radially deviated starting point, in the patients treated with centralization. This selection bias may be due to surgeons choosing between centralization and radialization on the basis of the intraoperative quality of the dorsoradial muscle mass and other soft tissues, reserving centralization for the patients who are more greatly affected. Microvascular second MTP joint transfer also provides a noticeably greater improvement in hand-forearm angle and improved “wrist” ROM compared with nonsurgical treatment, while achieving comparable ulnar length. Isolated soft-tissue release plus a bilobed flap offered only limited improvement in hand-forearm angle compared with nonsurgical treatment, but preserved “wrist” ROM and ulnar length. It is also notable that the reported final ulnar length was shorter after centralization than after other techniques. This may reflect a higher risk of premature fusion of the distal ulnar epiphysis with a more invasive surgical technique, or a selection bias as centralization was used instead of radialization in more severely affected cases.

These findings provide some support for current surgical practice, suggesting that in terms of the measured outcomes these operations “work,” but they do not of themselves show that a well-aligned, stabler “wrist” is more functional than a potentially unstable pseudarthrosis, or show whether the risks of surgery are justified. Until we have more robust, patient-centered data, that judgment is typically decided by the perceptions of the surgeon and the patient and parents regarding the balance of risks and benefits, and of the results of a technique in an individual surgeon’s hands.

Further unanswered questions include the management of the soft tissues and the ideal donor for microvascular reconstruction; are 2 short axial bones with a true joint (second MTP joint) best, or is a long bone such as the proximal aspect of the fibula better, even though it does not include a joint?

There are several major limitations to this study. The absence of randomized studies in the primary literature means that we cannot confidently ascribe differences in outcome to the techniques used, rather than to intrinsic differences between the patient groups. Most of the primary studies did not report individual patient data, or stratify their results by severity of the radial dysplasia. The scope for error is magnified as there is no standardization of the actual surgical technique among surgeons or centers globally, even for a named procedure (e.g., centralization). The details of patients’ perioperative care, splinting routine, age at treatment, and any subsequent procedures were variably reported, if they were reported at all. There was also little standardization of named outcomes, which may have used multiple different measurement techniques. Radiographic measurements are prone to errors of rotation and projection/magnification, as well as being dependent on the age and ossification stage of the child. This is inherent for a 2-dimensional measure of a 3-dimensional anomaly, in this case combining radial deviation and volar subluxation. Radiographic angles may also be affected by how cooperative a child is if the measurement is performed while he or she is awake, or by the muscle relaxant if it is performed intraoperatively. Detail regarding this information is seldom reported clearly. Clinical measurements of ROM are also limited; on their own, they do not clarify how useful or stable such movement is. Finally, as this study is a systematic review of the existing literature, it can only present the outcomes reported in that literature. There are ongoing studies of radial dysplasia^21^, and of congenital limb anomalies more broadly^29^, that will attempt to address which outcomes are important to patients by producing validated core outcome sets using patient panels.

These limitations mean that we must be cautious in our interpretation of these data. Nonetheless, they provide our current benchmark for comparing treatments, and the baseline for future research. Given the need for follow-up to skeletal maturity, and the relatively small numbers of patients seen annually even by national specialist centers, it is likely to be some time before we have large, methodologically robust studies to inform us. This review highlights the urgent need for this evidence to inform our practice. We would suggest that such studies will need multicenter international cooperation to power them adequately, and should focus on outcomes identified as relevant by patients and parents. Some of these should be measured using patient-reported tools, such as the PROMIS (Patient-Reported Outcomes Measurement Information System) Upper Extremity Score^30^. We appeal to congenital hand surgeons worldwide to engage with both the design and conduct of these studies.

## Appendix

Tables showing the characteristics and GRADE quality assessment of each included study as well as full data for each study (including additional references) are available with the online version of this article as a data supplement at jbjs.org (http://links.lww.com/JBJS/E480).
